# Supplementary material for: Development and validation of a quantitative, non-invasive, highly sensitive and specific, electrochemical assay for anti-SARS-CoV-2 IgG antibodies in saliva
Source: PLoS One. 2021 Jul 1;16(7):e0251342. doi: 10.1371/journal.pone.0251342 (PMC8248704; doi:10.1371/journal.pone.0251342)
Supplement: Attachment — Submitted filename: Response to Reviewers.docx [file pone.0251342.s001.docx]

To the Editor and Reviewers at PLOS ONE,

We thank the editors and reviewers for their time and feedback on our manuscript. Below we have listed our responses to the various comments given by the editor and reviewer: We have italicized comments by the editor/reviewers and provided our responses below each section.

**Editor Comments**

1. *Please ensure that your manuscript meets PLOS ONE's style requirements, including those for file naming. The PLOS ONE style templates can be found at*

[*https://journals.plos.org/plosone/s/file?id=wjVg/PLOSOne_formatting_sample_main_body.pdf*](https://urldefense.com/v3/__https:/journals.plos.org/plosone/s/file?id=wjVg*PLOSOne_formatting_sample_main_body.pdf__;Lw!!F9wkZZsI-LA!VYnk9LY4jPFLMncPDIEtplhbGPSFMQ1JJUA7mppg33zfWwoeG6lCQAEX305PjCL5rUY$) *and*

*https://journals.plos.org/plosone/s/file?id=ba62/PLOSOne_formatting_sample_title_authors_affiliations.pdf*

We have made modifications to follow the formatting guidelines outlined by the document.

*2. In your Methods section, please provide additional information about the participant recruitment method and the demographic details of your participants. Please ensure you have provided sufficient details to replicate the analyses such as:*

*a) the recruitment date range (month and year),*

*b) a description of any inclusion/exclusion criteria that were applied to participant recruitment,*

*c) a table of relevant demographic details,*

*d) a statement as to whether your sample can be considered representative of a larger population,*

*e) a description of how participants were recruited, and f) descriptions of where participants were recruited and where the research took place.*

We have included a “Participant Recruitment Method” section in our article.

*3. Please provide a sample size and power calculation in the Methods, or discuss the reasons for not performing one before study initiation. 4. To comply with PLOS ONE submission guidelines, in your Methods section, please provide additional information regarding your statistical analyses. For more information on PLOS ONE's expectations for statistical reporting, please see* [*https://journals.plos.org/plosone/s/submission-guidelines.#loc-statistical-reporting*](https://urldefense.com/v3/__https:/journals.plos.org/plosone/s/submission-guidelines.*loc-statistical-reporting__;Iw!!F9wkZZsI-LA!VYnk9LY4jPFLMncPDIEtplhbGPSFMQ1JJUA7mppg33zfWwoeG6lCQAEX305PtH-i1ys$)

We have included a description of the statistical methods and rationale for sample size used in our study in a section titled “Sample Size and Statistical Methods”

*5. Please provide the product number and any lot numbers of the ELISA kit purchased from for your study.*

We have included this in our methods and material section.

*6. Please note that PLOS does not permit references to “data not shown.” Authors should provide the relevant data within the manuscript, the Supporting Information files, or in a public repository. If the data are not a core part of the research study being presented, we ask that authors remove any references to these data.*

We have included figure 11 related to this description of stability.

*7. Please provide additional details regarding participant consent.*

*In the ethics statement in the Methods and online submission information, please ensure that you have specified what type you obtained (for instance, written or verbal, and if verbal, how it was documented and witnessed).*

*If your study included minors, state whether you obtained consent from parents or guardians.*

*If the need for consent was waived by the ethics committee, please include this information.*

We have amended this section in our manuscript.

*8b. Please also provide an updated Competing Interests Statement declaring this commercial affiliation along with any other relevant declarations relating to employment, consultancy, patents, products in development, or marketed products, etc.*

*Within your Competing Interests Statement, please confirm that this commercial affiliation does not alter your adherence to all PLOS ONE policies on sharing data and materials by including the following statement: "This does not alter our adherence to  PLOS ONE policies on sharing data and materials.” (as detailed online in our guide for authors* [*http://journals.plos.org/plosone/s/competing-interests).*](https://urldefense.com/v3/__http:/journals.plos.org/plosone/s/competing-interests).__;!!F9wkZZsI-LA!VYnk9LY4jPFLMncPDIEtplhbGPSFMQ1JJUA7mppg33zfWwoeG6lCQAEX305PWlrBAGA$)  *If this adherence statement is not accurate and  there are restrictions on sharing of data and/or materials, please state these. Please note that we cannot proceed with consideration of your article until this information has been declared.*

*We can confirm that this commercial affiliation does not alter our adherence to PLOS ONE polices on sharing data and materials. We have also included a statement of our interests in an attached cover letter*

*8c. Please include both an updated Funding Statement and Competing Interests Statement in your cover letter. We will change the online submission form on your behalf.*

*Please know it is PLOS ONE policy for corresponding authors to declare, on behalf of all authors, all potential competing interests for the purposes of transparency. PLOS defines a competing interest as anything that interferes with, or could reasonably be perceived as interfering with, the full and objective presentation, peer review, editorial decision-making, or publication of research or non-research articles submitted to one of the journals. Competing interests can be financial or non-financial, professional, or personal. Competing interests can arise in relationship to an organization or another person. Please follow this link to our website for more details on competing interests:* [*http://journals.plos.org/plosone/s/competing-interests*](https://urldefense.com/v3/__http:/journals.plos.org/plosone/s/competing-interests__;!!F9wkZZsI-LA!VYnk9LY4jPFLMncPDIEtplhbGPSFMQ1JJUA7mppg33zfWwoeG6lCQAEX305PKx1PnaM$)

We have included an updated cover letter to address #8.

*9. Please amend either the title on the online submission form (via Edit Submission) or the title in the manuscript so that they are identical.*

We have addressed this in our resubmission.

*10. Please review your reference list to ensure that it is complete and correct. If you have cited papers that have been retracted, please include the rationale for doing so in the manuscript text, or remove these references and replace them with relevant current references. Any changes to the reference list should be mentioned in the rebuttal letter that accompanies your revised manuscript. If you need to cite a retracted article, indicate the article’s retracted status in the References list and also include a citation and full reference for the retraction notice.*

We have reviewed the reference list.

**Reviewer Comments**

*Reviewer #1: The initial sentence of the abstract, and the rest of the abstract, do not clearly disclose how the assay works. What compound is electrochemically detected? It needs careful proofreading. E.g.,SARS-CoV-2 is misspelled in the abstract and Fig 9, and Line 57 should say “development *of* symptoms”.*

We thank the reviewer for noticing these issues. We have addressed these concerns and have amended our abstract/corrected these errors.

*Are the references in line 99 correct?*

We have added an additional reference (17) to correct this issue *24 washes are used in the assay, a rather large number. Were so many required?*

We thank the reviewer for their perceptiveness. Due to the early stage nature of the assay we have developed, we have settled for a more aggressive wash as a precaution. We expect in further development that the amount of wash buffer can be reduced.

 *With whom was the communication cited in line 186?*

This communication was made between our team and personnel at Orasure Technologies, Inc. the manufacturers of the saliva collector. We have modified our manuscript to reflect this.

*Some of the controls were 14 years old. Is there any evidence for the stability of antibodies in these samples?*

The observation of cross reacting IgG to receptor binding domain in the control samples demonstrates the stability of IgG molecules in these samples

*In lines 328 and 329, the receptor binding domain is described by 2 different incorrect names.*

Section omitted.

*The number of paired saliva and plasma samples is rather small. Have these numbers increased since the preprint or manuscript were prepared?*

At this time, our team has not aggressively pursued expanding our sample size. We believe our work’s context is as a preliminary demonstration of the viability of our approach.

*The impressively-measured saliva antibody levels seem high enough for standard ELISA.  Why use a specialized instrument?*

EFIRM has been developed specifically in the context of salivary diagnostics for a wide range of proteomic and genomic targets, and publications from our team have shown that the device has been particularly effective in minimizing interference/non-specific signal effects typically observed in ELISA of salivary samples.

*Reviewer #2: It is interesting work that could be useful to detect SARS-CoV-2 antibodies in saliva samples for COVID-19 diagnostics and for longitudinal monitoring of antibody levels in recovered patients. It is not entirely clear how easy/challenging sample processing/preparation is, what the level of reader complexity is, how long sample processing takes, etc. More details on underlying technology would be useful. Statements about theoretical “99.9999994%” specificity are somewhat dubious and not very meaningful.*

We thank reviewer #2 for their comments. We have expanded our introduction to include additional descriptions of the convenience of using the device and the ease of the assay.

In regards to the specificity value, we have calculated it based off of our reference range and cutoff values and believe it is compatible with existing calculation methods for specificity. We believe the body of our discussion however, does provide commentary to the nature and scope of both the analytical and clinical specificities so as to not mislead the scientific community.

**Conclusion**

We hope our edits/revisions to the manuscript have provided clarifications and improves to the original manuscript that are satisfactory. We are open to any additional feedback. We again thank the reviewers and editor for their time and efforts to our body of work.

Sincerely,

Charles M. Strom, M.D. PhD
